# Optimization and evaluation of Luminex performance with supernatants of antigen-stimulated peripheral blood mononuclear cells

**DOI:** 10.1186/s12865-016-0182-8

**Published:** 2016-11-11

**Authors:** Mathieu Surenaud, Céline Manier, Laura Richert, Rodolphe Thiébaut, Yves Levy, Sophie Hue, Christine Lacabaratz

**Affiliations:** 1 INSERM, U955, Equipe 16, Créteil, F-94010 France; 2 Université Paris Est, Faculté de médecine, Créteil, F-94010 France; 3 Vaccine Research Institute (VRI), Créteil, F-94010 France; 4 Université Bordeaux, ISPED, Centre INSERM U1219, F-33000 Bordeaux, France; 5 CHU de Bordeaux, pôle de santé publique, F-33000 Bordeaux, France; 6 INRIA SISTM, F-33405 Talence, France; 7 AP-HP, Hôpital H. Mondor – A. Chenevier, Service d’Immunologie Clinique et Maladies Infectieuses, F-94010 Créteil, France; 8 AP-HP, Hôpital H. Mondor – A. Chenevier, Service d’Immunologie Biologique, F-94010 Créteil, France

**Keywords:** Luminex, Cytokine, Reproducibility, Precision, Culture supernatant

## Abstract

**Background:**

The Luminex bead-based multiplex assay is useful for quantifying immune mediators such as cytokines and chemokines. Cross-comparisons of reagents for this technique from different suppliers have already been performed using serum or plasma but rarely with supernatants collected from antigen-stimulated peripheral blood mononuclear cells (PBMC). Here, we first describe an optimization protocol for cell culture including quantity of cells and culture duration to obtain reproducible cytokine and chemokine quantifications. Then, we compared three different Luminex kit suppliers.

**Results:**

Intraclass correlation coefficients (ICCs) for a 2-days stimulation protocol were >0.8 for IFNγ and Perforin. The specific concentration was maximal after two or five days of stimulation, depending on the analyte, using 0.5 million PBMC per well, a cell quantity that gave the same level of specific cytokine secretion as 1.0 million. In the second part of the study, Luminex kits from Millipore showed a better working range than Bio-Rad and Ozyme ones. For tuberculin purified protein derivative (PPD)-stimulated samples, the overall mean pooled coefficients of variation (CVs) for all donors and all cytokines was 17.2 % for Bio-Rad, 19.4 % for Millipore and 26.7 % for Ozyme. Although the different kits gave cytokine concentrations that were generally compatible, there were discrepancies for particular cytokines. Finally, evaluation of precision and reproducibility of a 15-plex Millipore kit using a “home-made” internal control showed a mean intra-assay CV <13 % and an inter-assay CV <18 % for each cytokine concentration.

**Conclusions:**

A protocol with a single round of stimulation but with two time points gave the best results for assaying different cytokines. Millipore kits appear to be slightly more sensitive than those from Bio-Rad and Ozyme. However, we conclude that the panel of analytes that need to be quantified should be the main determinant of kit selection. Using an internal control we demonstrated that a 15-plex magnetic Milliplex kit displayed good precision and reproducibility. Our findings should help optimize assays for evaluating immune responses during the course of disease or infection, or in response to vaccine or therapy.

**Electronic supplementary material:**

The online version of this article (doi:10.1186/s12865-016-0182-8) contains supplementary material, which is available to authorized users.

## Background

Cytokines and chemokines are soluble molecules playing key roles in the innate and adaptive immune responses. Quantifying these immune mediators has become increasingly important to obtain better descriptions of immune responses during the course of disease or infections, or to a vaccine, therapy and intervention; such detailed descriptions allow the identification of correlates of cure or protection. Various methods can be used to measure cytokines and chemokines, and enzyme-linked immunosorbent assay (ELISA) is still the most extensively employed [1]. This monoplex antibody-based immunoassay has been used for more than fifty years but can be laborious, sample consuming and expensive when studying profiles of large numbers of cytokines and chemokines. Therefore, a number of new technologies allowing simultaneous quantitative measurements of multiple cytokines and chemokines in small sample volumes have recently been developed. These multiplex immunoassay systems can be divided into two classes: planar assays and suspension microsphere assays [2]. The Multi-array Meso Scale Discovery (MSD) electro-chemiluminescence detection system (planar immunoassay) and Luminex xMAP technology (microsphere immunoassay) are considered to be the two most suitable platforms for biomarker analysis or quantification [3–5].

The growing use of Luminex technology has led to different kits being made available by several companies, and we decided to work with this multiplex platform. The Luminex system allows the analysis of up to 100 analytes in a single well. Relatively small volumes (25–50 μl) of serum, plasma, other body fluids, or cell culture supernatant can be assayed for cytokines and chemokines. Extensive data have been published validating the Luminex platform for detection of multiple analytes, by comparing this technique with ELISA [6–12]. Cross-comparisons between reagent suppliers showed variability in absolute cytokine concentration results in serum or plasma [13–15]. Moreover, a study comparing Luminex kits from three different companies, including World Health Organization (WHO) cytokine standards in addition to the standards provided with each kit, revealed striking differences in results for cytokine concentrations in serum [16].

There have been few studies comparing different Luminex kits using supernatants from antigen-stimulated cell culture, an approach used in vaccine research. One study compared Luminex kits from three different suppliers for the determination of cytokine levels in whole blood culture supernatant [17], and two studies compared cytokine and chemokine levels in supernatants from antigen specific-stimulated peripheral blood mononuclear cells (PBMC) as tested with different commercial Luminex kits [18, 19]. These studies revealed differences of performance (accuracy, recovery, reproducibility) between the suppliers tested. Defawe and colleagues published a study on optimization and validation of a Luminex protocol to assess cytokine and chemokine production by vaccine-specific cells to allow better characterization of immune responses [20]. However, the optimization of the “cell culture step” described in their work could be improved. The aim of the present study was to optimize a cell culture protocol (including quantity of cells, culture duration, and positive control) to obtain reproducible cytokine and chemokine quantifications by Luminex technology in antigen-stimulated PBMC supernatants; the second aim was then to compare the performances of different Luminex kits.

## Results

### Reproducibility of the Luminex assay

We first performed an intra-laboratory reproducibility study with a multiplex assay developed and shared by the Baylor Institute for Immunology Research (BIIR) and using polystyrene beads (Table 1). PBMC from six Human Immunodeficiency virus (HIV)-infected patients (CD4 count > 500 mm3, viral load < 50 copies/ml) were cultured without stimulation or with: (i) LIPO-5 HIV-1 vaccine [21], (ii) a mix of five long HIV-1 peptides (LP mix), (iii) a 15-mers HIV-1 peptide pool and (iv) Staphylococcal Enterotoxin B (SEB) as a positive control. Three independent cultures were performed: 1 million PBMC/well were cultured in triplicate for 2 and 11 days, and assayed for IFNγ, perforin, IL-5 and IL-17. In the 11 day-cultures (two-step stimulation protocol), high concentrations and large variations were observed for all cytokines with SEB and peptide stimulations (Additional file 1: Figure S1). For example, LP mix induced from 4,810 to 357,392 pg/ml for IFNγ with PBMC from patient A and from 16 to 5,421 pg/ml for perforin with PBMC from patient B. By contrast, a short stimulation period without re-stimulation could lead to more reproducible results (Additional file 2: Figure S2). At day 2, SEB strongly stimulated the PBMC, and IL-17 (the lower secreted cytokine) measurement seemed to be the most variable (24 to 66 pg/ml for patient A and 43 to 135 pg/ml for patient B). HIV-specific stimulation induced very low concentrations of IL-5 and IL-17, but the LP mix resulted, in samples from patient A, in an IFNγ concentration from 2,932 to 12,091 pg/ml and a Perforin concentration of 3,403 to 4,511 pg/ml. As expected, intraclass correlation coefficients (ICCs) for HIV-stimulated samples for the 11-day stimulations were low (Table 2) whereas good ICCs (>0.8) were observed with the 2-day cultures for IFNγ (using either FI or concentrations) and Perforin (using concentration in range only). We excluded extrapolated concentration values, i.e., below or above the limits of quantification (LOQ) and corresponding fluorescent intensity (FI). Nevertheless, even excluding extrapolated values, ICCs were not in all cases acceptable. For IL-5, the agreement was too low (ICC < 0.6).

**Table 1:** Description of Luminex kits

Supplier	Kit name	Type of beads	Number of analytes	Analytes	Part of the study
BIIR + Biolegend	-	Polystyrene	4	IFNγ, IL-5, IL-17 + Perforin	Reproducibility
Millipore	Milliplex MAP Human Cytokine (#SPR137)	Magnetic	11	IL-17, IL-21, IP-10, IFNγ, IL-10, IL-13, IL-1β, IL-2, IL-5, IL-6, TNFα	Kinetics of cytokine production + Quantity of stimulated PBMC
Millipore	Milliplex MAP Human Cytokine (#SPR171)	Polystyrene	10	IL-17, IL-21, IFNγ, IL-10, IL-13, IL-1β, IL-2, IL-5, IL-6, TNFα	Kinetics of cytokine production + Quantity of stimulated PBMC
Millipore	Milliplex MAP Human Th17	Magnetic	10	IL-17A, IL-22, IFNγ, IL-10, IL-13, IL-1β, IL-2, IL-5, IL-6, TNFα	Kinetics of cytokine production + Quantity of stimulated PBMC
Ozyme	Procarta Immunoassays kit (Affymetrix)	Magnetic	16	GM-CSF, IFNγ, IL-10, IL-12p40, IL-13, IL-17A, IL-1β, IL-2, IL-22, IL-5, IL-6, IP-10, MCP-1, MIP-1α, MIP-1β, TNFα	Comparison of commercial Luminex Kits
Millipore	Milliplex MAP Human Cytokine (#SPR158) + Milliplex MAP Human Cytokine/Chemokine	Magnetic	15 + 1	GM-CSF, IFNγ, IL-10, IL-13, IL-17A, IL-1β, IL-2, IL-22, IL-5, IL-6, IP-10, MCP-1, MIP-1α, MIP-1β, TNFα + IL-12p40	Comparison of commercial Luminex Kits
Bio-Rad	Bio-plex Pro assays + Bio-Plex Pro Human Th17 Cytokine assays	Magnetic	28 + 1	IL-1β, IL-1ra, IL-2, IL-4, IL-5, IL-6, IL-7, IL-8, IL-9, IL-10, IL-12p70, IL-13, IL-15, IL-17A, Eotaxin, Basic FGF, G-CSF, GM-CSF, IFNγ, IP-10, MCP-1, MIP-1α, MIP-1β, PDGF-BB, RANTES, TNFα, VEGF, and IL-12p40 + IL-22	Comparison of commercial Luminex Kits
Millipore	Milliplex MAP Human Th17	Magnetic	15	GM-CSF, IFNγ, IL-10, IL-17 F, IL-13, IL-17A, IL-1β, IL-2, IL-22, IL-9, CCL20/MIP-3α, IL-5, IL-23, IL-27, and TNFα	Internal QC study

**Table 2:** Intraclass correlation coefficients (ICCs) across HIV-stimulated samples

Cytokine		FI			Conc^a^ (pg/ml)			Conc in range (pg/ml)		
	Culture duration	Samples (n)	Total meas. (n)	ICC	Samples (n)	Total meas. (n)	ICC	Samples (n)	Total meas. (n)	ICC
IFNγ	11d	16	114	.69	16	107	.11	16	81	.51
Perforin	11d	16	91	.66	16	71	.17	16	61	.72
IL-5	11d	16	114	.36	16	106	.25	16	99	.23
IL-17	11d	16	114	.02	16	69	.02	16	28	.19
IFNγ	2d	17	135	**.93**	17	129	**.88**	17	122	**.81**
Perforin	2d	17	111	.16	17	97	NA	17	95	**.88**
IL-5	2d	17	135	.58	17	91	.55	17	86	.53
IL-17	2d	17	135	.43	17	37	.29	1	1	**-**

ICCs were calculated across 9 replicate cultures. The combination ‘donor x stimulation’ was treated as one sample. *d* day, *Conc* concentration, *meas* measurement. ICCs > 0.8 are indicated in bold

^a^including extrapolated values, NA: G matrix ‘not positive definite’

### Kinetics of cytokine production

We performed kinetic studies over 7 days with samples from two healthy donors to determine optimal culture duration for quantification of weakly secreted cytokines in stimulated-PBMC supernatants. First, we found that the culture duration (without rIL-2 supplemented medium) should not be longer than 6 days to maintain cell viability above 70 % using non-stimulated or tuberculin purified protein derivative (PPD)-stimulated PBMC (Additional file 3).

**Fig. 1:**
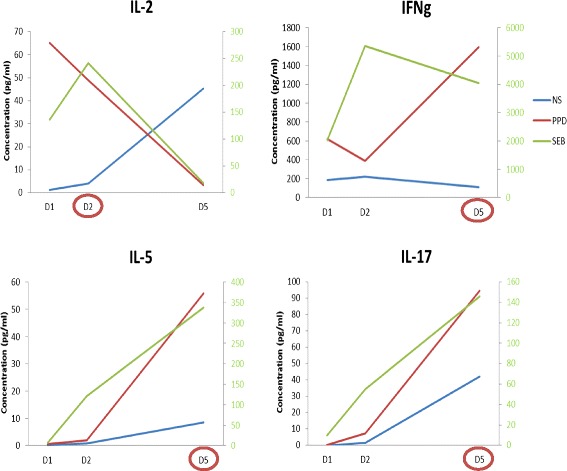
Kinetics of cytokine production. Milliplex kits from Millipore (see Material and Methods for details) were used to perform kinetic experiments with PBMC from two healthy donors stimulated for 1, 2 or 5 days with PPD (1 μg/ml) and SEB (10 ng/ml) as positive control. Non-stimulated (NS) PBMC were used as negative control. Results obtained for samples from donor 1 (IL-2 and IL-17) and donor 2 (IFNγ and IL-5) are shown. NS and PPD values correspond to the left y axis and SEB values to the right y axis

T cell subsets can be characterized according to their cytokine secretion profile. We focused on four cytokines: the T-helper (Th)1 cytokines IFNγ and IL-2, the Th2 cytokine IL-5, and the Th17 cytokine IL-17. The optimal culture duration was defined as that giving the highest specific concentration (i.e. the largest positive difference between stimulated and non-stimulated cells). The specific concentration of IL-2 was maximal after two days of stimulation whereas the specific concentration peaks for IFNγ, IL-5 and IL-17 were on day 5 (Fig. 1). These findings suggested that different time points (here day 2 and day 5) are required for optimal quantifications of different cytokines in supernatants from antigen-stimulated PBMC.

### Number of stimulated PBMC

**Fig. 2:**
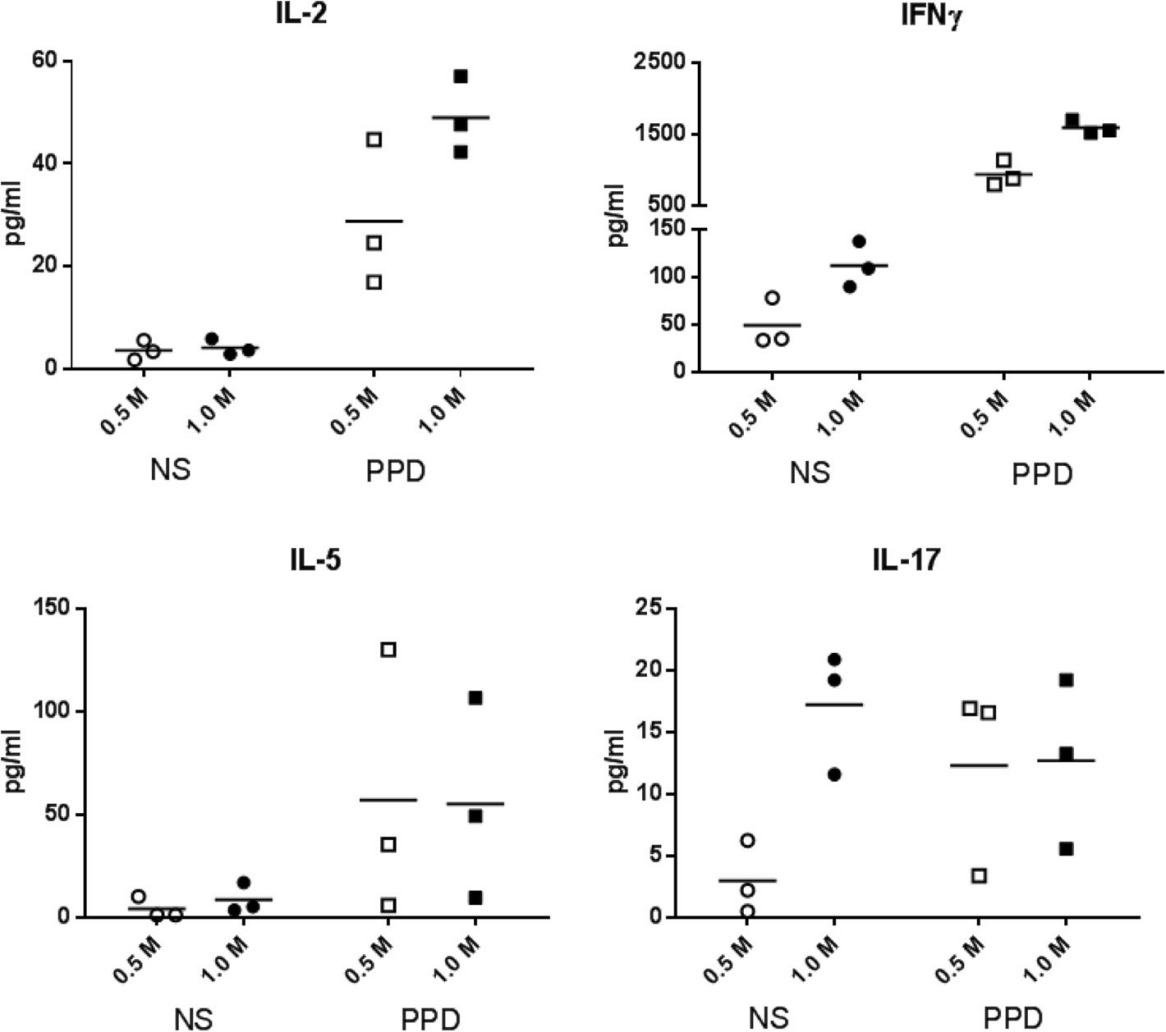
Optimization of cell concentration. Milliplex kits from Millipore (see Material and Methods) were used to perform cell concentration optimization experiments using 0.5 or 1.0 million Non-Stimulated (NS) or PPD-Stimulated PBMC from two healthy donors cultured in triplicate for 2 or 5 days. Results obtained with samples from donor 1 (IL-2 and IL-17) and donor 2 (IFNγ and IL-5) are shown at the optimal time point defined for each cytokine. Mean cytokine concentrations are represented by black lines

The quantity of PBMC available in clinical trials and the numbers of specific cells are often small, so we compared the use of 0.5 and 1.0 million cells per well: PBMC from two healthy donors were stimulated with PPD and assayed for the same four cytokines at their optimal time point (Fig. 2). The concentration of IL-2 was slightly lower after the stimulation of 0.5 million of PBMC per well than 1.0 million of PBMC/well (29 vs. 49 pg/ml) without any difference in the background (4 pg/ml). The concentration of IFNγ was also slightly lower with 0.5 million than 1.0 million stimulated PBMCs (942 vs. 1,596 pg/ml), but a similar relative difference was observed for the background (49 vs.112 pg/ml). There was no significant difference in IL-5 concentrations between the two densities of PBMC. The concentration of IL-17 following PPD stimulation was similar at the two densities of PBMC but the background was significantly lower at 0.5 million PBMCs per well (3 vs. 17 pg/ml). Thus, the stimulation of PBMC at 0.5 and 1.0 million per well resulted in relatively similar levels of specific cytokine secretion (i.e. background subtracted).

### Comparison of three commercial Luminex kits

Next, we compared Luminex kits supplied by different manufacturers (Millipore, Ozyme and Bio-Rad). We first analysed mean % CV from standard and samples coming from polystyrene bead based 10-plex and magnetic bead based 15-plex kits and found that they were lower with magnetic beads (3.1 and 15.5, respectively) compared to polystyrene ones (5.3 and 25.1, respectively), thus confirming the better precision of magnetic kits as previously described [19]. In the next experiments, only Luminex kits using magnetic beads were selected and analytes were quantified at their optimal time point previously defined.

We first compared vendor-reported (theoretical) working range (lower limit of quantification (LLOQ) and upper limit of quantification (ULOQ)) and experimental working range for each kit. The working range was defined as that for which the observed concentration was between 70 % and 130 % of expected concentration using the five parameter log-logistic (5PL) standard curve. The 16 cytokines common to the three kits were studied and the results are summarized in Table 3. A sensitivity lower than that reported by the vendor was observed for some analytes for all kits. The LLOQ was particularly discordant with Ozyme and Bio-Rad kits for GM-CSF and especially with the Bio-Rad kit for IL-12p40 (302.6 pg/ml instead of 15.5 pg/ml, a three dilution difference). Ozyme and Bio-Rad kits also showed a narrower standard spectrum than expected: the observed ULOQ diverged by one or two dilutions from the theoretical value for many cytokines. Overall, we obtained a better working range with Millipore than Ozyme and Bio-Rad kits.

**Table 3:** Working range and sensitivities

Analyte	Ozyme				Millipore				Bio-rad			
	theoretical		experimental		theoretical		experimental		theoretical		experimental	
	LLOQ	ULOQ	LLOQ	ULOQ	LLOQ	ULOQ	LLOQ	ULOQ	LLOQ	ULOQ	LLOQ	ULOQ
IL-17A	2.92	47900	2.90	12124^a^	9.60	30000	1.91	29561	1.78	29157	1.73	24822
IP-10	1.35	22100	1.10	5328^a^	1.60	5000	7.75^a^	4999	3.50	57400	3.12	52465
MCP-1	2.95	48400	2.60	12076^a^	1.60	5000	8.29^a^	5034	1.52	24920	1.64	24492
MIP-1α	0.52	8550	0.52	534^b^	1.60	5000	8.33^a^	5455	1.20	19707	1.21	3630^a^
MIP-1β	6.56	107500	24.58^a^	108640	1.60	5000	7.42^a^	4979	0.85	13960	1.03	10114
GM-CSF	4.29	70300	59.10^b^	70495	50.00	130000	10.00	117900	1.24	20314	16.27^b^	20993
IFNγ	0.20	3200	0.20	2323	8.00	25000	1.81	24966	2.13	34920	7.50^a^	34918
IL-10	0.34	5550	0.27	5424	2.40	7500	0.40	7502	1.59	26094	1.56	6551^a^
IL-13	2.89	47400	2.68	48745	6.40	20000	1.37	20019	2.40	39338	2.43	9065^a^
IL-22	13.47	220700	13.56	13752^b^	24.00	75000	4.66	75275	4.19	68313	4.22	17087^a^
IL-1β	0.71	11700	0.71	10791	4.80	15000	1.00	15029	1.90	31130	1.91	6893^a^
IL-2	0.40	6550	1.25^a^	7119	9.60	30000	1.92	30166	0.84	13690	3.09^a^	14356
IL-5	0.84	13800	3.28^a^	16674	5.60	17500	1.20	17052	2.34	38356	2.35	2392^b^
IL-6	0.92	15000	0.83	14549	3.20	10000	0.53	10233	1.54	25160	1.56	23428
TNFα	2.05	33600	1.87	2099^b^	3.20	10000	0.68	9999	6.54	107172	6.78	26507^a^
IL-12p40	3.34	54700	3.28	3092^b^	3.20	10000	15.46^a^	9965	4.72	77321	302.60^c^	77346

Differences between theoretical and experimental limits of quantifications (LOQs) expressed in pg/ml are highlighted with ^a^, ^b^, and ^c^ for 1-dilution, 2-dilution and 3-dilution differences from theoretical LOQ, respectively

We then used intra-assay coefficients of variation (CVs) to compare the precision of the three kits with standards and samples. All kits performed well for standard intra-assay precision: the mean of standard intra-plate CVs for the 16 common cytokines was below 8 % for all kits (Additional file 4: Figure S3). As this work was initially performed to select the best Luminex kit for a TB/HIV study, PBMC from two healthy donors, one HIV-1-positive patient and one tuberculosis (TB)-positive patient were cultured in triplicate for 2 and 5 days without antigen (NS) or with PPD, early secreted antigenic target 6 kDa protein (ESAT-6) or SEB. The supernatant of each culture well was considered to be one sample for Luminex analysis. Not all the cytokines were included in the final analysis because their values were above the ULOQ (MCP-1, MIP-1α, MIP-1β and IL-6) or below the LLOQ (IL-12p40). Eleven common cytokines were thus analyzed: IP-10, IL-10, IL-1β and IL-2 after 2-day culture and IL-5, GM-CSF, IFNγ, IL-17A, IL-13, IL-22, and TNFα after 5-day culture. CVs of three culture replicates for these 11 cytokines were plotted against the mean concentration for NS, PPD, SEB and ESAT-6 stimulations. A characteristic non-linear relationship was observed for all kits (Additional file 5: Figure S4): the CV rose substantially as the mean value approached zero.

**Fig. 3:**
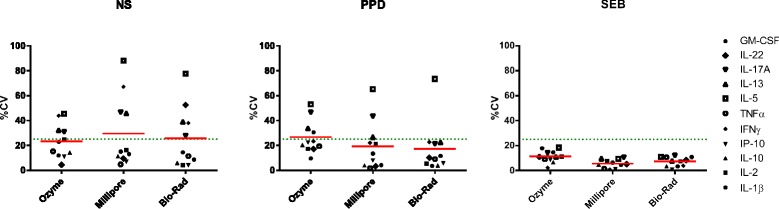
Intra-assay precision of samples. %CV of the concentrations (mean CVs for four donors calculated using triplicate cultures for each cytokine) as determined with Luminex magnetic kits from Ozyme, Millipore, and Bio-Rad for the 11 common analyzed cytokines. Left panel: Non-stimulated PBMC; middle panel: PPD-stimulated PBMC; right panel: SEB-stimulated PBMC. Mean CVs calculated for all donors and all cytokines are represented by red lines

Figure 3 shows %CVs obtained for each kit according to culture conditions — NS, PPD- and SEB-stimulated PBMC — highlighting the generally accepted 25 % cutoff value for biomarker assay precision [22]. For SEB stimulations, the overall mean pooled CV for all donors and all cytokines (calculated from triplicates per cytokine and per patient) was between 1 and 18 %. For PPD stimulations, the values were 17.2 % for Bio-Rad, 19.4 % for Millipore and 26.7 % for Ozyme; only the IL-5 CV was above the 25 % threshold with the Bio-Rad kit in contrast to Millipore and Ozyme kits which showed CV >25 % for IL-5, IL-13 and IL-17A. Applying a 30 % CV threshold like the study by Defawe and colleagues [20], the Millipore kit gave acceptable %CVs for IL-13. The mean pooled CV was even higher for NS samples: 23.4 % for Ozyme, 25.8 % for Bio-Rad and 29.5 % for Millipore.

**Fig. 4:**
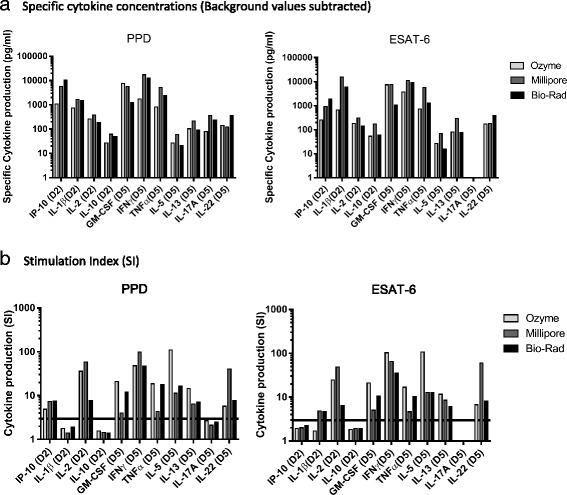
Cytokine profiles in TB-infected patient. Cytokine production by PPD- and ESAT-6-stimulated PBMC from a TB-infected donor was measured with kits from Millipore, Ozyme and Bio-Rad. **a** Specific cytokine Concentrations (background values subtracted). **b** Stimulation Index (ratio between stimulated and background concentration values). The value for IL-22 from non-stimulated PBMC given by the Millipore kit was inferior to the range of the standard curve and was therefore imputed by the lowest extrapolated value for this cytokine (3.1 pg/ml). The IL-17A concentration with ESAT-6 stimulation was a null value for all kits, so SI could not be calculated. The positive threshold for the SI was set at 3 (black line)

Next, we analyzed cytokine concentrations in the supernatant from TB-infected stimulated PBMC. The kits yielded different cytokine concentrations with quality control samples (QCs) from Millipore and these experiments also revealed some cross-reactivities for the Ozyme kit: it detected IL-22 at a high concentration in the Millipore QCs which do not contain this cytokine (Additional file 6). Although the kits gave results that were generally compatible, there were discrepancies between them for particular cytokines (Fig. 4a). Another approach to measuring cytokine production is the use of a stimulation index (SI), calculated as a ratio between mean antigen-stimulated PBMC concentration and mean non-stimulated PBMC concentration. Like Defawe and colleagues [20], we used a SI threshold of 3 for positivity and observed that the kits tested gave divergent results for IL-1β (Fig. 4b). Although no discrepancy between the kits for positive or negative SI was observed for the other cytokines, some SIs differed by 10 to 100 between kits. Thus, our study showed variability in the measurement of cytokine production (either with absolute concentration or SI) in supernatants from antigen-stimulated PBMC with luminex kits coming from different manufacturers.

### Internal QC for Luminex study

For the last experiment, we used the Millipore kit because: 1) its working range and sensitivity were better than the two other kits tested, 2) %CVs were as good as the other kits, 3) no cross-reactivity was observed, 4) it was the only kit with QCs provided, and 5) it contains most analytes that we were interested in.

**Fig. 5:**
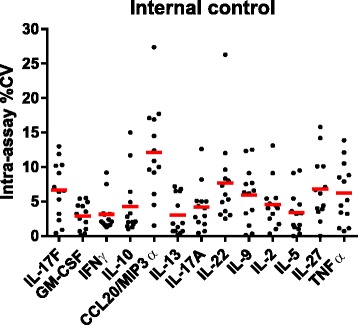
Intra-assay variability of internal control. %CV concentration for each of the 13 plates calculated using internal control duplicates for the selected 15-plex Millipore magnetic kit (see Material and Methods). Mean %CVs are represented by red lines. IL-23 and IL-1β %CVs were not calculated due to concentration values < LLOQ and > ULOQ, respectively

A “home-made” internal control (culture supernatant from SEB-stimulated PBMC) was used in addition to the two Millipore QCs (high and low concentration) to evaluate intra- and inter-assay precision and thus to check the reproducibility of the assay. This internal control was used to determine variations observed for a control sample prepared and stored under the same conditions as our study samples. Intra- and inter-assay %CVs for the internal control assayed in duplicate are reported in Fig. 5 and Table 4, respectively, for 13 of the 15 cytokines (IL-23 and IL-1β %CVs were not calculated for the internal control due to concentration values < LLOQ and > ULOQ, respectively). Low intra-assay variability was observed both with the internal control and Millipore QCs. Mean CVs for each cytokine were <13 % for the “home-made” internal control (Fig. 5) and <7 % for Millipore QCs (Additional file 7). Only two of the 169 calculated intra-assay CVs for the internal control were above the 25 % threshold. The inter-assay variability was also low. The CV was <18 % for each cytokine concentration in the internal control (Table 4), and <14 % in the Millipore QCs (Additional file 7). Thus, the Millipore Milliplex kits demonstrated a good precision and reproducibility in our hands.

**Table 4:** Inter-assay variability of internal control

Plates	IL-17F	GM-CSF	IFNγ	IL-10	CCL20/MIP3α	IL-13	IL-17A	IL-22	IL-9	IL-2	IL-5	IL-27	TNFα
1	2091.2	7423.2	5300.1	81.4	9523.6	2248.3	74.0	45.4	225.6	89.8	191.9	808.9	3424.9
2	2059.3	6734.8	6833.8	82.0	9009.3	2537.1	90.9	65.0	256.9	107.0	224.2	1081.6	4126.7
3	1963.3	6811.0	5144.7	74.7	5706.4	2268.3	82.2	46.3	216.1	90.9	214.7	786.4	3571.6
4	2215.3	8326.4	5348.7	78.4	8241.1	2412.0	83.1	53.8	231.1	104.7	205.1	1308.3	3953.4
5	1998.1	6735.9	5506.4	61.1	8311.7	2064.6	72.1	48.5	195.9	89.2	172.9	1026.8	3385.1
6	2238.5	7407.3	6040.5	85.1	6113.7	2259.9	89.1	61.5	263.3	84.2	218.3	1093.6	4302.2
7	2473.7	8788.0	5809.2	71.3	8211.6	2560.0	88.3	47.0	278.8	98.5	243.6	1102.3	4128.4
8	2425.1	7440.4	5180.8	81.6	10147.7	2429.5	84.7	50.8	254.4	105.9	214.3	1190.2	4310.1
9	1945.9	6719.6	4545.8	68.4	8901.7	2384.8	82.4	53.3	198.0	95.8	221.2	1175.7	4241.0
10	2165.8	7817.6	7062.7	88.5	10767.6	2729.8	87.8	50.3	296.4	110.3	241.2	1070.5	5285.1
11	2185.4	6660.9	4907.7	68.6	8756.7	2387.7	70.6	39.6	180.1	91.9	170.5	914.5	3409.2
12	2151.4	7223.0	5796.1	65.0	8429.9	2304.6	71.1	42.6	224.8	86.8	195.3	1088.9	4515.4
13	2231.2	7964.7	5324.4	78.5	7028.9	2618.0	87.1	51.7	237.8	113.9	230.2	1249.2	4951.5
%CV	7.4	9.2	12.8	11.0	17.2	7.4	9.0	14.0	14.4	10.0	11.0	14.6	14.3

Mean concentration values of duplicate Internal Controls (pg/ml) and %CVs of mean concentration calculated using the 13 inter-plates internal control mean concentration values for the selected 15-plex Millipore magnetic kit (see Material and Methods section for detailed analytes). IL-23 and IL-1β %CVs were not calculated due to concentration values < LLOQ and > ULOQ, respectively

## Discussion

The Luminex platform is widely used to study immune responses but only few studies evaluated its reproducibility and/or compared kits from different suppliers using supernatants from stimulated PBMC [18–20]. This work was designed to evaluate reproducibility of cytokine quantification by Luminex assay in supernatants from antigen-stimulated PBMC and to improve this quantification by optimization of the culture protocol and eventually the choice a particular commercial kit. We used cryopreserved PBMC because such samples are typical of those used in clinical trials. Moreover, it has been shown that using validated standard operating procedures (SOPs) for isolation, cryopreservation and thawing of PBMC, CD4^+^ and CD8^+^ cells maintain full functionality in cytokine enzyme-linked immunospot (ELISpot) assays [23]. We first tested two different stimulation protocols: a two-round stimulation (i.e. cytokine measurement on day 11, after a first stimulation on day 0 and a re-stimulation on day 9) in the presence of rIL-2 supplemented medium, and a one-round stimulation for 1 to 7 days without rIL-2 supplemented medium. The two-round stimulation protocol induced a lot of variability. However, this type of protocol could be valuable to investigate “on/off” signals if no precise quantification of secreted cytokines is required. We also observed some variability, although to a lesser extent, with the 48 h stimulation protocol. Like ELISpot assay, the observed variability with the Luminex assay, which is inherent to cell culture, probably resulted in part from variations in counting and pipetting, and low antigen-specific cell frequency [24]. One way to minimize this variability is to perform multi-well stimulations and to pool supernatants before assaying them by the Luminex platform.

Another factor to take into consideration for cytokine quantifications in stimulated-PBMC supernatants is the cell viability. Defawe et al. claimed that a high cell viability limited the potential effect of dead cells on cytokine content measured by Luminex [20]. However, we observed that having cell viability above 80 % on the day of stimulation (D0) was not sufficient to ensure low cell mortality during the culture. Indeed, a dose of 100 ng/ml SEB (10-fold lower than that used in the study by Defawe and colleagues) killed near 50 % of PBMC after 5 days of culture (data not shown). Consequently, the antigen dose needs to be adapted.

Our results suggest that two different time-points are required for optimal quantifications of different cytokines (IFNγ, IL-2, IL-5 and IL-17) in supernatants from antigen-stimulated PBMC. This is consistent with published results. Indeed, differences in the kinetics of IFNγ and IL-2 production have already been observed in whole blood cell cultures [25]. The duration of in vitro stimulation of PBMC with recall antigens determines the subset distribution of IFNγ-producing cells, and 4 or 5 days of stimulation are necessary to have a predominance of IFNγ-secreting T cells [26]. Lagrelius and colleagues reported that 3 and 7 days are the most suitable periods for stimulation to measure various cytokine levels in whole blood supernatant [27]. Furthermore, our results validating the use of 0.5 million PBMC per well are in agreement with those obtained by others with cytomegalovirus (CMV) stimulation [20].

After setting up our protocol for cytokine measurements in supernatants coming from antigen-stimulated PBMC, we compared results obtained with Luminex magnetic kits coming from three different manufacturers. We observed a lower sensitivity than that reported by the vendor for some analytes for all kits but overall, we obtained a better working range with Millipore than Ozyme and Bio-Rad kits. Also, Djoba Siawaya and colleagues reported a comparison between commercial Luminex cytokine kits using whole blood supernatant and concluded that the LINCO (now Millipore) kit was the most sensitive for measuring concentrations of numerous recombinant cytokines in samples that had been spiked with serial dilutions of the standards provided by the manufacturer [17]. Consistent with our results, Belabani and colleagues showed that a Bio-Rad magnetic Luminex kit was not as sensitive as suggested by the manufacturer for some cytokines [28]. In their condensed performance-validation strategy for multiplex detection kits used in studies of human clinical samples recently published, these authors proposed a complete validation of Luminex assays. Among the critical points, we would also recommend 1) extending the standard curve to include lower values to check the working range and true sensitivity; 2) including LLOQs and ULOQs as validation samples to validate their concentration; 3) excluding values below the LLOQ or using specific statistical analyses that takes into account left-censoring [29]; 4) diluting and reanalyzing all samples with a concentration above the ULOQ; and 5) fixing the thresholds of %CV and %Recovery at 25 % (30 % for LLOQs) as acceptance criteria for samples, as defined by Lee and colleagues [22].

We then compared the precision of the three kits with standards and samples. It is important to note that we used here triplicate cultures in our Luminex assays (and not supernatant pooled from several culture wells) to estimate the variability cytokine assays results, and this could explain at least in part the high variability observed. As observed for ELISpot [23], but also tetramer assays and cytokine flow cytometry [24], the relationship between CV and mean response level is not linear. The stability of the CV is poor at very low mean values, and antigen-specific assays are often used to analyze data in a range where CV is non-linear. Consequently, Maecker and colleagues suggested using Standard Deviation (SD), which may be related linearly to the response mean, to compare the precision of different assays [24]. However, we did not find a clear linear correlation between SD and mean concentration (Additional file 8), and therefore we used %CV to compare precision of the different kits. Our results confirmed that the lower the secretion, the higher the %CV and that IL-5, IL-13 and IL-17A were difficult to quantify accurately even at the optimal time point, whatever the kit used. Another group has compared Luminex kits for the measurement of cytokines in supernatants collected from antigen-stimulated PBMC: they report good intra-plate but poor inter-plate precision of the Bio-Rad 17-plex kit [18] and found that the Millipore magnetic kit gave the most reproducible results [19]. In our hands, the good precision of Millipore kits was confirmed, as mean % CV of standards and samples from 2 different kits (10-plex versus 15-plex magnetic beads based kits) were similar.

We also used a “home-made” internal control to evaluate precision and reproducibility of Milliplex kits as suggested by De Jager and colleagues in their work with serum, plasma and whole blood culture supernatant to define the prerequisites for cytokine measurements in clinical trials with multiplex immunoassays [30]. We showed that the Millipore Milliplex kits demonstrated a good precision and reproducibility in our hands. Similarly, in their study of optimization and qualification of a multiplex bead array to assess cytokine and chemokine production by vaccine-specific cells, Defawe and colleagues found CVs for the intra-assay and inter-day variability below 30 % for all but one analyte with a Millipore kit [20].

Our study showed variability in the measurement of absolute cytokine concentration in supernatants from antigen-stimulated PBMC with Luminex kits coming from different manufacturers. A previous study showing that a Bio-Rad kit was more sensitive than an Invitrogen kit revealed the potential for differences in absolute cytokine concentration reported by these two kits [18]. Other studies also showed variability in the measurement of absolute cytokine concentrations in serum or plasma [13–16]. Possibly, the different capture and detection antibodies are the major causes of the differences in performance of multiplex kits from different manufacturers as well as the heterogeneity in analytes within each kit. Also, differences between the purified recombinant proteins used to generate the standard curves, and between assay buffers supplied by the manufacturers are likely to contribute to the divergent results, as well as cross-reactivities observed with the Ozyme kit. Thus, the main criterion to select a manufacturer should be the availability of the panel of analytes that needs to be quantified and mixing data coming from different kits for analyses should be banned.

To determine positivity of samples, Defawe and colleagues used an empirical method based on their experience using the assay and they aimed to control the false positive rate below 3 % [20]. The first criterion was “minimum analyte concentration over background” determined for each analyte and the second criterion was “minimum fold increase over background concentration”. The minimum fold increase over background concentration criterion was set as three-fold for all analytes. We used the same second criterion in this study to calculate the SI, but the definition used for the first criterion is debatable because there is no consensus formula to determine the minimum analyte concentration over background.

## Conclusions

Here, we demonstrate that a protocol with a single round of stimulation but with two time points (day-2 and day-5) gave the optimal quantifications for different cytokines using 0.5 million PBMC per well, a cell quantity that gave the same level of specific cytokine secretion as 1.0 million. Millipore kits appear to be slightly more sensitive than those from Bio-Rad and Ozyme. However, we conclude that the panel of analytes that need to be quantified should be the main determinant of kit selection and that the same kit must be used throughout any given study. We also think that it is important to use the same batch number throughout the study to allow results to be compared between plates. To ensure the validity of the results, we also recommend the use of an internal control. Using this type of sample, we demonstrated that a 15-plex magnetic Milliplex kit displayed good precision and reproducibility. Moreover, our study suggests that particular attention should be paid to cell viability during cell culture to ensure that high cell mortality does not perturb the nature of cytokines secreted. Finally, to limit the variability inherent to cell culture, we suggest the use of supernatant pooled from at least three culture replicates to avoid large variations in the cytokine concentrations observed. In view of the cost of the technique and the good reproducibility of the results obtained with Millipore QC and with our internal control (intra- and inter-plate %CVs), it is unclear whether supernatant replicates are beneficial if standard curves are performed in duplicate and are extended to include lower levels so as to confirm the working range. In summary, our findings should help optimize assays for quantifying immune mediators during the course of disease or infection, or in response to vaccine or therapy.

## Methods

### Blood sample processing

Whole blood specimens were collected from two healthy controls (from the *Etablissement Français du Sang*, Créteil, France), seven HIV-seropositive patients (from the Mondor Hospital cohort, Créteil, France) and one TB-infected patient (from St-Louis Hospital, Paris, France).. PBMC were isolated using LSM 1077 density gradient media (PAA), washed with PBS without calcium or magnesium (Life Technologies), frozen in fetal calf serum (FCS, PAA) containing 10 % DMSO (Sigma) and stored in liquid nitrogen. For each experiment, about 12-18x10^6^ PBMC (one cryovial) were thawed in R-20 (RPMI 1640 + glutamax containing 100 U/ml penicillin and 0.1 mg/ml streptomycin (Life Technologies) and 20 % FCS) medium and rested for 3 h at 37 °C, under 5 % CO_2_ in R-10HS (RPMI 1640 + glutamax containing 100U/ml penicillin and 0.1 mg/ml streptomycin and 10 % AB Human serum (HS, PAA)) medium. A minimal cell viability of 75 % as measured with an Auto 2000 cellometer (Nexcelom) after resting was required for use.

### PBMC stimulations

Two different protocols were used for PBMC stimulations. For the first one (2-step stimulation), PBMC (1×10^6^) were stimulated for 2 days at 37 °C, under 5 % CO_2_ in a final volume of 300 μl R-10HS in 96 deep well plates (Greiner) with (i) 2 μg/ml of LIPO-5 vaccine composed of the clade B LAI HIV-1 Nef 66–97, Nef 116–145, Gag 17–35, Gag 253–284 and Pol 325–355 regions, coupled to a lipid tail [21]; (ii) 2 μg/ml of a mix of five long HIV-1 peptides (LP mix, NeoMPS) corresponding to the sequences included in the LIPO-5 vaccine without the lipid tail; (iii) 2 μg/ml of a pool of 36 HIV-1 15-mer peptides (NeoMPS) overlapping by 11 amino acids and spanning Nef, Gag and Pol sequences encoded by the LIPO-5 vaccine. Aliquots of 200 μl of supernatant were harvested on day 2, and 900 μl of fresh R-10HS medium containing 100 IU/ml human recombinant IL-2 (rIL-2, Miltenyi) was added. On days 4, 7 and 8, 500 μl of medium was replaced with fresh R10-HS medium containing 100 IU/ml rIL-2. On day 9, 800 μl of supernatant was discarded, cells were transferred in 96 V-bottom wells culture plates (Greiner), washed in PBS, resuspended in R-10HS medium containing the same concentrations of the corresponding antigens and incubated for 2 days at 37 °C, under 5 % CO_2_ in a final volume of 200 μl R-10HS. Positive and negative controls consisted of PBMC cultured with 100 ng/ml of SEB (Sigma, only for the two last days of stimulation) and PBMC cultured in R-10HS alone (non-stimulated, NS), respectively. For the second protocol of PBMC stimulation (1-step stimulation), PBMC (0.5×10^6^ or 1×10^6^) were stimulated for 1 to 7 days at 37 °C, under 5 % CO_2_ in a final volume of 300 μl R-10HS in 96 deep well plates (Greiner) with: (i) 2 μg/ml of LIPO-5 vaccine [21]; (ii) 2 μg/ml of LP mix (NeoMPS); (iii) 2 μg/ml of a pool of 36 HIV-1 15-mer peptides (NeoMPS) overlapping by 11 amino acids; (iv) 1 μg/ml of PPD (Statens Institute) and (v) 5 μg/ml of ESAT-6, a *Mycobacterium tuberculosis* T-specific antigen (Statens Institute). Positive and negative controls consisted of PBMC cultured with 10 ng/ml of SEB (Sigma) and PBMC cultured in R-10HS alone (non-stimulated, NS), respectively. Supernatants were harvested at days 2/11 for the re-stimulation (2-step) protocol and after various times of culture for the 1-step protocol. They were then frozen at -80 °C until assessment.

### Cytokine and chemokine assays with Luminex kits

All Luminex kits used for this study are described in detail in Table 1. Luminex reproducibility was evaluated using a Luminex kit developed and shared by the Baylor Institute for Immunology Research (BIIR, Dallas, TX, USA). This kit contained polystyrene beads from BIIR and Biolegend and standards from Biolegend. We used three Milliplex MAP kits from Millipore to optimize our stimulation protocol: a 11-plex human cytokine magnetic bead panel kit (#SPR137), a 10-plex human cytokine panel kit (#SPR171), and a 10-plex human Th17 magnetic bead panel. We compared the following commercial kits: one 16-plex magnetic Procarta Immunoassays kit (Affymetrix) from Ozyme; one 15-plex Milliplex MAP human cytokine magnetic bead panel kit (#SPR158) and one simplex Milliplex MAP human cytokine/chemokine magnetic bead panel, both from Millipore; one Bio-Plex Pro assays magnetic kit and one Bio-Plex Pro human Th17 cytokine assays kit, both from Bio-Rad. A 15-plex Milliplex MAP human Th17 magnetic bead panel (Millipore, Saint-Quentin en Yvelines, France) was selected for the last part of our study; precision was evaluated using intra- and inter-variability with Millipore QCs and an internal control (pool of SEB-stimulated PBMC supernatants from the two healthy donors). All samples were acquired on a Bioplex-200 instrument (Bio-Rad, Marnes-la-Coquette, France). All experiments were performed by the same operator according to the manufacturers’ instructions.

### Statistical analysis

The concentration of each analyte was obtained by interpolating FI to a dilution standard curve over at least 7 dilution points supplied with the kit and calculated using a 5PL curve by the Bio-Plex Manager 5.0 software (Bio-Rad). ICCs were used to assess reproducibility. The ICC is a measure of reliability, assessing the proportion of overall variability that is explained by the variability due to differences between samples [31]. Generally, reliability is considered to be good if the point estimate of the intra-class correlation coefficients is at least >0.7, and ideally > 0.8. ICCs were derived from linear mixed effects models with a random effect for the intercept. The ICC was defined as the ratio of variance between supernatants to total variance. The assumption of Gaussian distributions of the residuals was verified. CVs (standard deviation over mean) were used to assess precision. A SI (calculated as a ratio between stimulated-cell supernatant concentration values and background concentration values) was used to reveal differences between the kits. All graphs were prepared using Microsoft Excel and GraphPad Prism. SAS (version 9.2, SAS Institute, Cary, North Carolina, USA) and GraphPad Prism (version 6.0, GraphPad Software, La Jolla, California, USA) were used for statistical analyses.

## Additional files

Additional file 1: Figure S1. Intra-laboratory reproducibility with 11-day cultures. Results after 11-day cultures of PBMC from two representative HIV-infected patients (A and B, CD4 count > 500 mm3, HIV-1 RNA < 50 copies/ml) stimulated with LIPO-5 vaccine, a mix of 5 long HIV-1 peptides (LP mix), a pool of 15-mers HIV-1 peptides, or SEB as a positive control. IFNγ, IL-5, and IL-17 polystyrene beads from Baylor Institute for Immunology Research, Texas, USA and Perforin beads from Biolegend were used. Cultures were performed on three different days (black, green, blue) in triplicate for each day with 1.0 million PBMC per well. (PDF 110 kb)
Additional file 2: Figure S2. Intra-laboratory reproducibility with 2-day cultures. Results after 2-day cultures of PBMC from two representative HIV-infected patients (A and B, CD4 count > 500 mm3, HIV-1 RNA < 50 copies/ml) stimulated with LIPO-5 vaccine, a mix of 5 long HIV-1 peptides (LP mix), a pool of 15-mers HIV-1 peptides, or SEB as a positive control. IFNγ, IL-5, and IL-17 polystyrene beads from Baylor Institute for Immunology Research, Texas, USA and Perforin beads from Biolegend were used. Cultures were performed on three different days (black, green, blue) in triplicate for each day with 1.0 million PBMC per well. (PDF 110 kb)
Additional file 3: PBMC viability over 7 days of culture. PBMC Viability (%) from one healthy donor for two different experiments was determined by Acridine Orange/Propidium iodide (AOPI) staining with Auto 2000 cellometer (Nexcelom) with NS-, PPD (1 μg/ml)-, or SEB (10 or 100 ng/ml)-stimulated PBMC for 1 to 7 days. (PDF 184 kb)
Additional file 4: Figure S3. Intra-assay precision of standards. %CVs concentration calculated using standard duplicates from Luminex magnetic kits from Ozyme, Millipore and Bio-Rad for the 16 cytokines common to all three kits. Mean %CVs for all cytokines are represented by red lines. (PDF 27 kb)
Additional file 5: Figure S4. Non-linear relationship between %CV and mean cytokine concentration. %CVs concentration (mean CVs for four different donors and four conditions [NS, PPD, SEB and ESAT-6 stimulations] calculated using culture triplicates for each of the 11 cytokines analyzed) plotted against corresponding mean concentrations for Ozyme, Millipore and Bio-Rad kits. (PDF 98 kb)
Additional file 6: IL-22 cross-reactivity with Ozyme kit. IL-22 concentration (pg/ml) determined with the three different kits in Millipore QC that do not contain this cytokine. (PDF 6 kb)
Additional file 7: Intra- and inter-assay precision for Millipore QCs. %CV concentration for each of the 13 plates calculated using Millipore QCs (QC1 = high and QC2 = low) duplicates for the selected 15-plex Millipore magnetic kit (see Material and Methods). IL-23 and IL-1β %CVs were not calculated due to concentration values < LLOQ and > ULOQ, respectively. Mean concentration values of Millipore QCs (QC1 = high and QC2 = low) duplicates (pg/ml) and %CVs of mean concentration calculated using the 13 inter-plates mean concentration values for the selected 15-plex Millipore magnetic kit (see Material and Methods section for detailed analytes). IL-23 and IL-1β %CVs were not calculated due to concentration values < LLOQ and > ULOQ, respectively. (PDF 252 kb)
Additional file 8: Absence of linear correlation between SD and mean concentration values. SD calculated using culture triplicates for NS, PPD- and SEB-stimulated PBMC from TB-infected patient plotted against IFNγ, IL-5 and IL-17A corresponding mean concentration values for Ozyme, Millipore and Bio-Rad kits. (PDF 14 kb)

## Abbreviations

5PL: Five parameter log-logistic
CMV: Cytomegalovirus
CV: Coefficient of variation
ELISA: Enzyme-linked immunosorbent assay
ELISpot: Enzyme-linked immunospot
ESAT-6: Early secreted antigenic target 6 kDa protein
FI: Fluorescence intensity
HIV: Human immunodeficiency virus
ICC: Intraclass correlation coefficient
LLOQ: Lower limit of quantification
LOQ: Limit of quantification
LP: Long peptides
NS: Non-stimulated
PBMC: Peripheral blood mononuclear cells
PPD: Tuberculin purified protein derivative
QC: Quality control
SD: Standard deviation
SEB: Staphylococcal enterotoxin B
SI: Stimulation index
TB: Tuberculosis
Th: T-helper
ULOQ: Upper limit of quantification
