# Supplementary figures and images for: Optimization and evaluation of Luminex performance with supernatants of antigen-stimulated peripheral blood mononuclear cells
Source: BMC Immunol. 2016 Nov 11;17:44. doi: 10.1186/s12865-016-0182-8 (PMC5106791; doi:10.1186/s12865-016-0182-8)

A

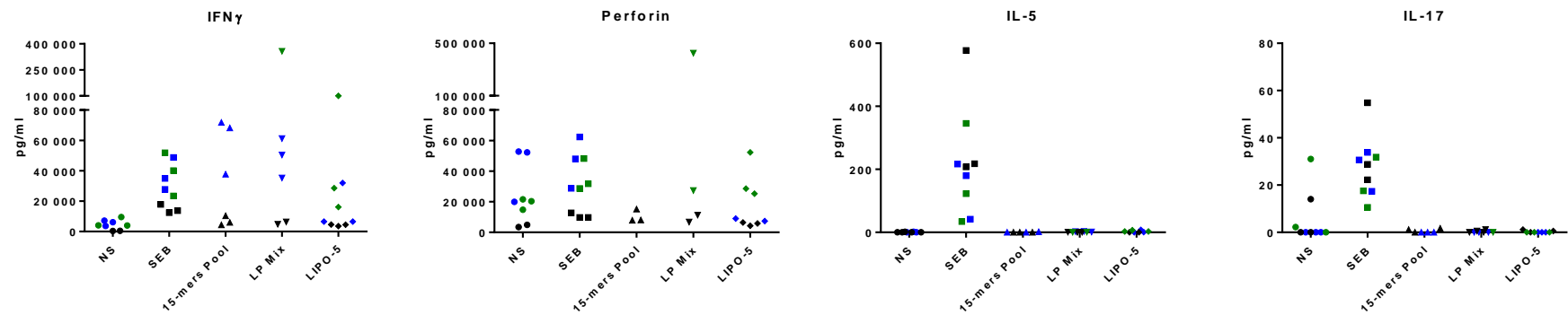

B

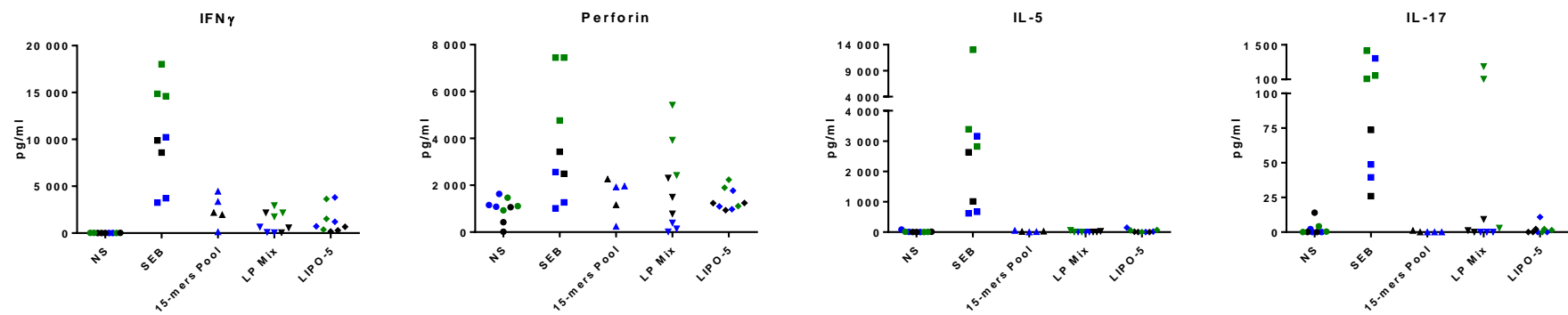

Supplement: Additional file 1: Figure S1. — Intra-laboratory reproducibility with 11-day cultures. Results after 11-day cultures of PBMC from two representative HIV-infected patients (A and B, CD4 count > 500 mm3, HIV-1 RNA < 50 copies/ml) stimulated with LIPO-5 vaccine, a mix of 5 long HIV-1 peptides (LP mix), a pool of 15-mers HIV-1 peptides, or SEB as a positive control. IFNγ, IL-5, and IL-17 polystyrene beads from Baylor Institute for Immunology Research, Texas, USA and Perforin beads from Biolegend were used. Cultures were performed on three different days (black, green, blue) in triplicate for each day with 1.0 million PBMC per well. (PDF 110 kb) [file 12865_2016_182_MOESM1_ESM.pdf]

A

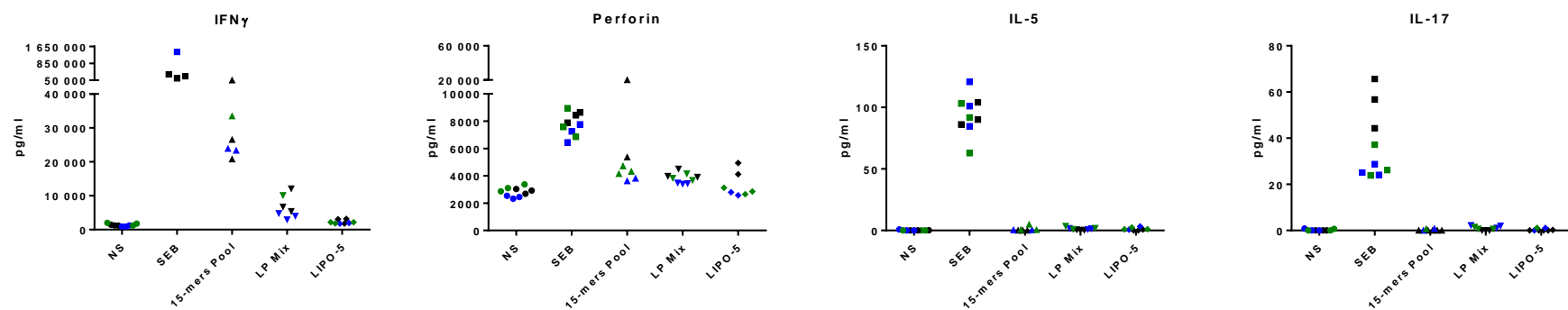

B

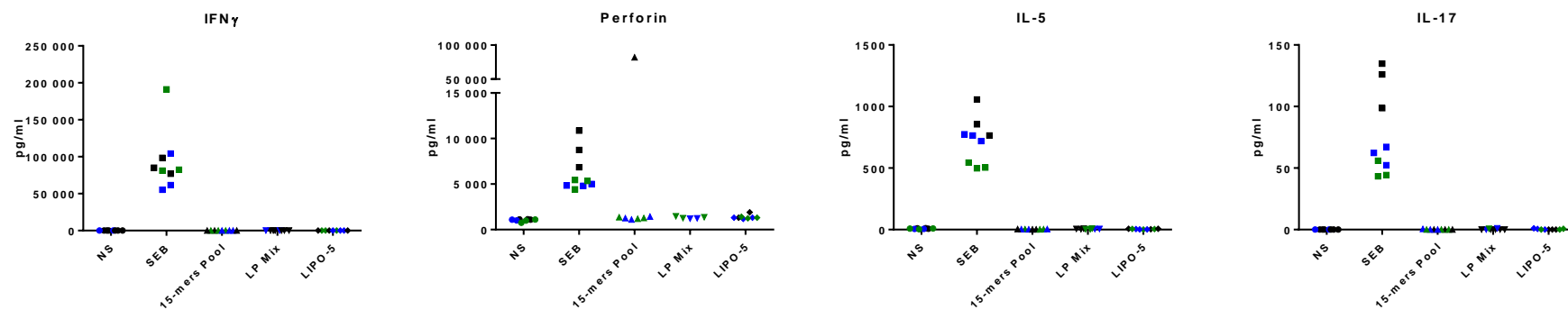

Supplement: Additional file 2: Figure S2. — Intra-laboratory reproducibility with 2-day cultures. Results after 2-day cultures of PBMC from two representative HIV-infected patients (A and B, CD4 count > 500 mm3, HIV-1 RNA < 50 copies/ml) stimulated with LIPO-5 vaccine, a mix of 5 long HIV-1 peptides (LP mix), a pool of 15-mers HIV-1 peptides, or SEB as a positive control. IFNγ, IL-5, and IL-17 polystyrene beads from Baylor Institute for Immunology Research, Texas, USA and Perforin beads from Biolegend were used. Cultures were performed on three different days (black, green, blue) in triplicate for each day with 1.0 million PBMC per well. (PDF 110 kb) [file 12865_2016_182_MOESM2_ESM.pdf]

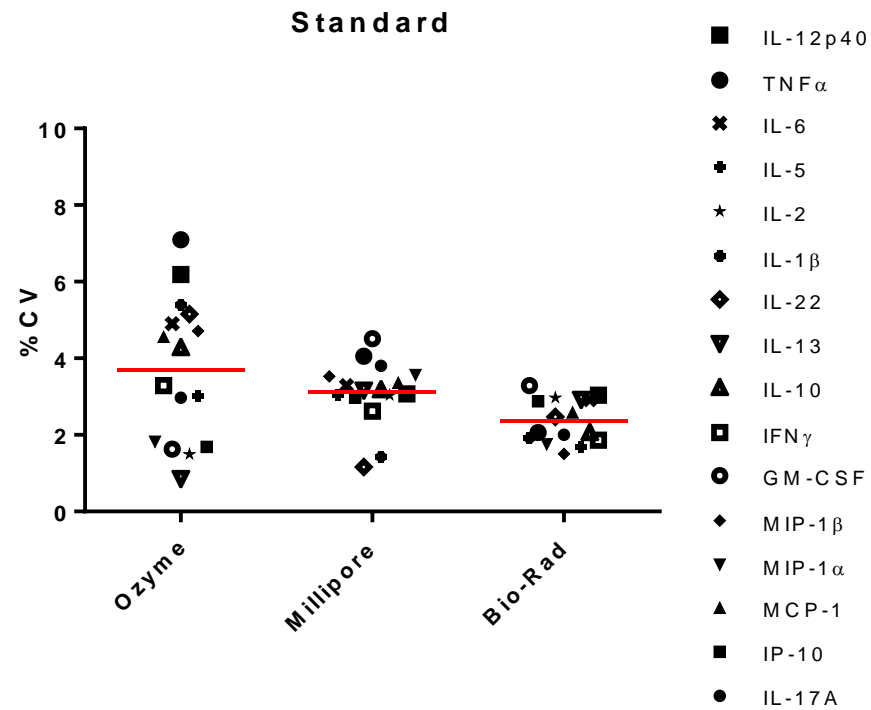

Supplement: Additional file 4: Figure S3. — Intra-assay precision of standards. %CVs concentration calculated using standard duplicates from Luminex magnetic kits from Ozyme, Millipore and Bio-Rad for the 16 cytokines common to all three kits. Mean %CVs for all cytokines are represented by red lines. (PDF 27 kb) [file 12865_2016_182_MOESM4_ESM.pdf]

Ozyme

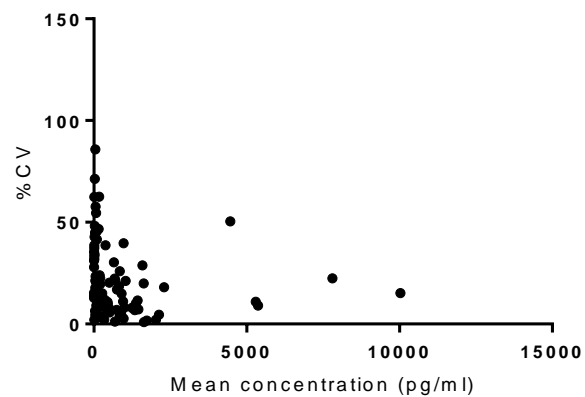

Millipore

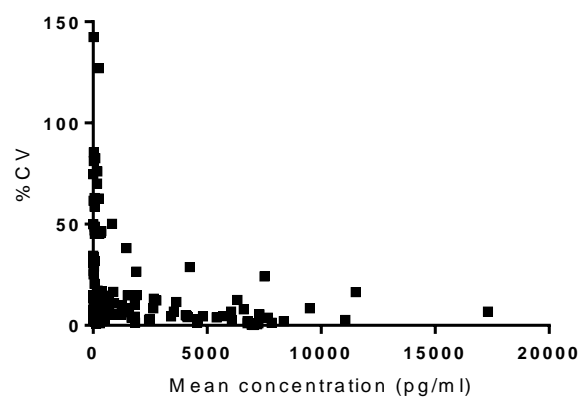

Bio-Rad

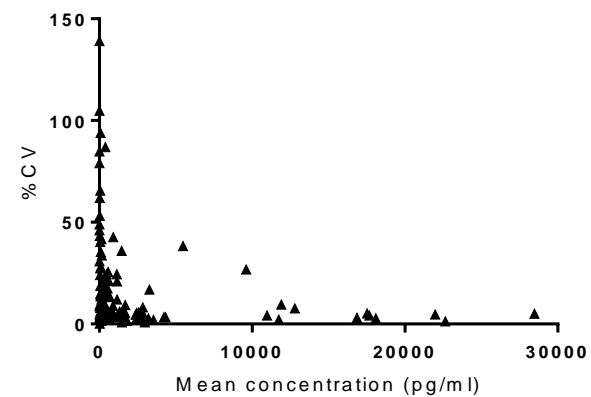

Supplement: Additional file 5: Figure S4. — Non-linear relationship between %CV and mean cytokine concentration. %CVs concentration (mean CVs for four different donors and four conditions [NS, PPD, SEB and ESAT-6 stimulations] calculated using culture triplicates for each of the 11 cytokines analyzed) plotted against corresponding mean concentrations for Ozyme, Millipore and Bio-Rad kits. (PDF 98 kb) [file 12865_2016_182_MOESM5_ESM.pdf]

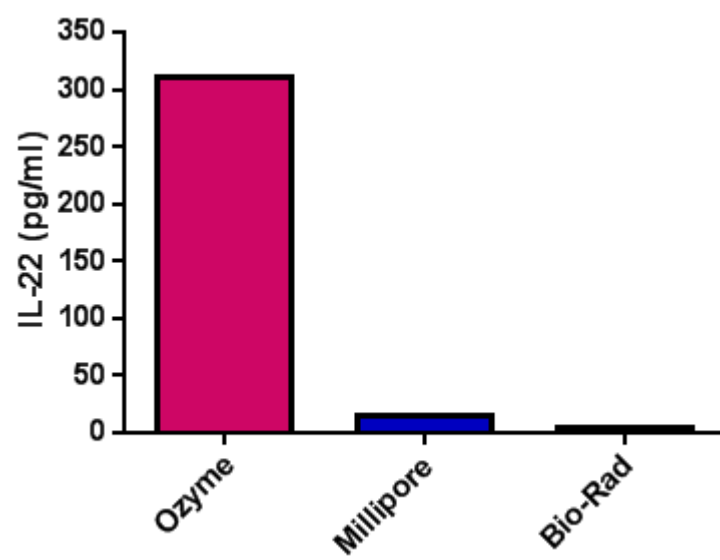

Supplement: Additional file 6: — IL-22 cross-reactivity with Ozyme kit. IL-22 concentration (pg/ml) determined with the three different kits in Millipore QC that do not contain this cytokine. (PDF 6 kb) [file 12865_2016_182_MOESM6_ESM.pdf]

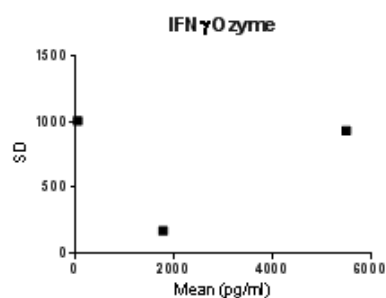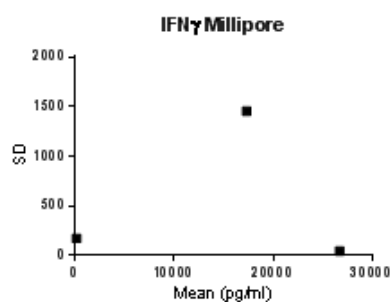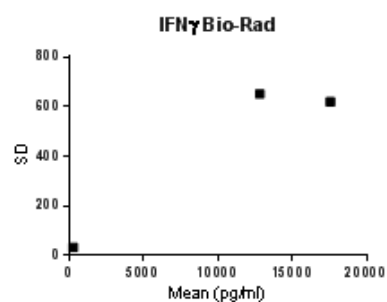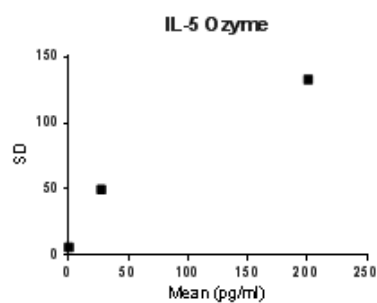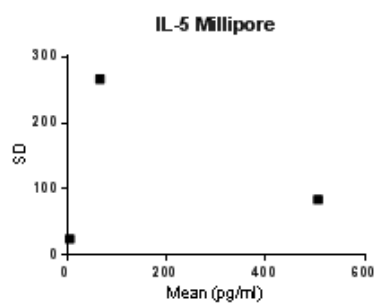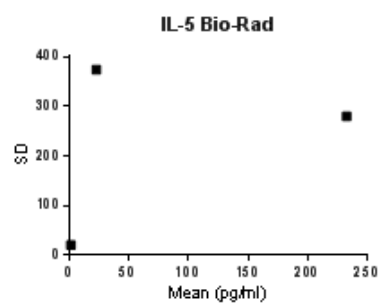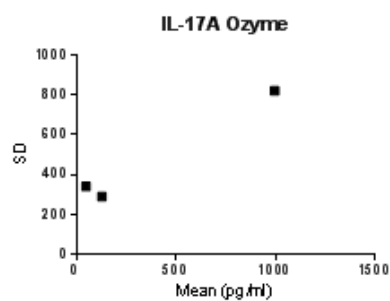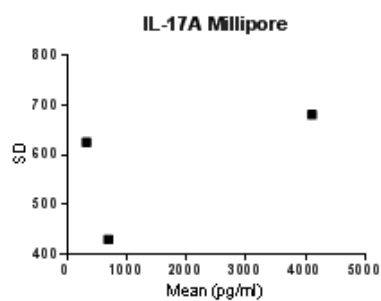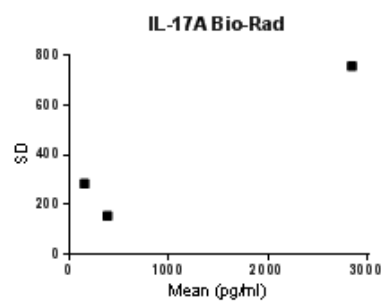

Supplement: Additional file 8: — Absence of linear correlation between SD and mean concentration values. SD calculated using culture triplicates for NS, PPD- and SEB-stimulated PBMC from TB-infected patient plotted against IFNγ, IL-5 and IL-17A corresponding mean concentration values for Ozyme, Millipore and Bio-Rad kits. (PDF 14 kb) [file 12865_2016_182_MOESM8_ESM.pdf]
