# Supplementary material for: Optimization and evaluation of Luminex performance with supernatants of antigen-stimulated peripheral blood mononuclear cells
Source: BMC Immunol. 2016 Nov 11;17:44. doi: 10.1186/s12865-016-0182-8 (PMC5106791; doi:10.1186/s12865-016-0182-8)
Supplement: Additional file 3: — PBMC viability over 7 days of culture. PBMC Viability (%) from one healthy donor for two different experiments was determined by Acridine Orange/Propidium iodide (AOPI) staining with Auto 2000 cellometer (Nexcelom) with NS-, PPD (1 μg/ml)-, or SEB (10 or 100 ng/ml)-stimulated PBMC for 1 to 7 days. (PDF 184 kb) [file 12865_2016_182_MOESM3_ESM.pdf]

PBMC viability (%)

			D1	D2	D3	D4	D5	D6	D7
First experiment	Healthy donor 1 (1,0 million cells per well)	NS	89,4	83,3	82,5	80,1	77,9	75,6	**63,4**
First experiment	Healthy donor 1 (1,0 million cells per well)	PPD (1µg/ml)	92,7	83,8	81,3	76,8	74,7	76,4	**68,2**
First experiment	Healthy donor 1 (1,0 million cells per well)	SEB (**100ng/ml**)	86,8	79,4	71,6	**57,1**	**38,9**	**37,0**	**31,5**
Second experiment	Healthy donor 1 (1,0 million cells per well)	NS	82,3	82,7	ND	ND	78,6	ND	ND
Second experiment	Healthy donor 1 (0,5 million cells per well)	NS	75,0	76,9	ND	ND	79,3	ND	ND
Second experiment	Healthy donor 1 (1,0 million cells per well)	PPD (1µg/ml)	82,1	82,8	ND	ND	78,5	ND	ND
Second experiment	Healthy donor 1 (0,5 million cells per well)	PPD (1µg/ml)	74,9	75,0	ND	ND	74,1	ND	ND
Second experiment	Healthy donor 1 (1,0 million cells per well)	SEB (**100ng/ml**)	79,4	79,4	ND	ND	**60,3**	ND	ND
Second experiment	Healthy donor 1 (0,5 million cells per well)	SEB (**100ng/ml**)	74,1	75,8	ND	ND	**67,2**	ND	ND
Second experiment	Healthy donor 1 (1,0 million cells per well)	SEB (10ng/ml)	88,2	82,4	ND	ND	76,8	ND	ND
Second experiment	Healthy donor 1 (0,5 million cells per well)	SEB (10ng/ml)	74,8	79,3	ND	ND	71,7	ND	ND

ND: Not Deternined
