# Supplementary material for: Optimization and evaluation of Luminex performance with supernatants of antigen-stimulated peripheral blood mononuclear cells
Source: BMC Immunol. 2016 Nov 11;17:44. doi: 10.1186/s12865-016-0182-8 (PMC5106791; doi:10.1186/s12865-016-0182-8)
Supplement: Additional file 7: — Intra- and inter-assay precision for Millipore QCs. %CV concentration for each of the 13 plates calculated using Millipore QCs (QC1 = high and QC2 = low) duplicates for the selected 15-plex Millipore magnetic kit (see Material and Methods). IL-23 and IL-1β %CVs were not calculated due to concentration values < LLOQ and > ULOQ, respectively. Mean concentration values of Millipore QCs (QC1 = high and QC2 = low) duplicates (pg/ml) and %CVs of mean concentration calculated using the 13 inter-plates mean concentration values for the selected 15-plex Millipore magnetic kit (see Material and Methods section for detailed analytes). IL-23 and IL-1β %CVs were not calculated due to concentration values < LLOQ and > ULOQ, respectively. (PDF 252 kb) [file 12865_2016_182_MOESM7_ESM.pdf]

Intra-assay variability

	Plates	IL-17F	GM-CSF	IFNγ	IL-10	CCL20/MIP3α	IL-13	IL-17A	IL-22	IL-9	IL-2	IL-5	IL-27	TNFα	
		Obs Conc %CV	Obs Conc %CV	Obs Conc %CV	Obs Conc %CV	Obs Conc %CV	Obs Conc %CV	Obs Conc %CV	Obs Conc %CV	Obs Conc %CV	Obs Conc %CV	Obs Conc %CV	Obs Conc %CV	Obs Conc %CV	
	1	0,9	0,5	2,3	7,8	2,1	1,3	2,0	6,7	0,1	3,5	0,8	5,0	4,4	
	2	2,6	2,8	6,3	5,6	1,6	9,2	4,7	1,0	0,8	2,3	1,0	0,2	17,8	
	3	8,5	5,9	2,6	5,0	5,8	9,4	8,3	7,6	12,6	8,6	9,2	9,2	8,9	
	4	7,5	11,3	9,5	12,6	7,9	9,1	8,9	13,7	6,4	8,1	6,8	13,2	9,2	
	5	2,9	2,6	1,4	2,4	1,5	4,0	6,4	4,7	1,7	8,1	3,4	6,0	1,5	
	6	1,3	4,9	1,2	7,2	4,0	4,8	1,8	5,6	5,3	2,4	6,6	5,7	2,6	
**QC1**	7	3,8	2,1	0,6	4,3	0,2	0,7	2,1	10,4	8,5	0,8	2,9	0,3	1,1	
	8	0,3	0,4	5,6	3,7	0,7	2,2	0,6	0,9	3,1	1,8	4,5	1,3	4,4	
	9	10,9	5,0	2,1	8,4	6,2	6,8	3,6	4,5	9,3	11,3	6,8	8,1	6,2	
	10	8,6	7,1	3,3	4,6	6,6	2,9	2,1	5,9	2,9	4,9	8,2	0,7	1,9	
	11	9,1	0,9	5,6	9,6	2,2	4,2	1,6	0,9	3,2	3,1	2,1	0,9	2,0	
	12	1,6	6,5	4,7	3,2	5,8	3,5	0,1	2,9	8,2	0,5	0,2	5,1	4,3	
	13	1,7	0,9	4,2	5,1	4,0	0,4	4,2	5,5	5,4	2,1	7,2	2,4	3,3	
	**Pooled %CV**	**4,6**	**3,9**	**3,8**	**6,1**	**3,7**	**4,5**	**3,6**	**5,4**	**5,2**	**4,4**	**4,6**	**4,5**	**5,2**	**4,6**
	1	4,7	5,3	1,7	1,9	3,6	4,2	3,3	4,5	11,4	2,3	4,2	0,2	0,1	
	2	0,8	1,5	2,0	0,8	3,0	3,0	0,7	0,3	1,4	2,7	0,7	0,5	1,8	
	3	1,0	7,5	1,5	5,4	1,9	3,2	3,6	1,7	2,4	1,1	3,1	1,1	4,9	
	4	4,9	5,2	1,6	2,4	0,1	0,9	2,8	9,5	2,9	5,9	0,8	1,7	2,1	
	5	2,1	7,3	0,6	7,3	2,7	1,0	1,2	4,4	2,6	8,5	4,1	6,4	3,7	
	6	1,0	4,3	3,3	0,6	0,2	0,4	4,8	3,7	3,3	14,3	3,2	2,9	1,9	
**QC2**	7	4,7	0,4	1,5	3,2	0,5	3,1	1,8	0,5	2,8	1,3	4,0	4,5	0,6	
	8	16,3	18,3	4,4	10,9	9,9	4,9	14,8	**25,7**	16,8	14,0	6,3	9,9	11,7	
	9	6,4	0,8	6,6	5,0	4,0	0,0	2,0	0,8	0,3	0,9	3,3	4,4	0,0	
	10	5,9	3,5	0,4	7,0	2,6	2,9	2,6	6,2	1,8	5,0	5,7	0,7	2,4	
	11	1,2	1,5	6,6	8,0	1,1	1,8	2,0	4,2	9,7	3,5	2,2	5,0	5,3	
	12	3,8	1,5	1,8	0,3	2,1	1,6	1,4	3,5	3,2	3,6	2,3	3,4	1,5	
	13	2,5	3,5	2,4	1,9	0,1	3,3	2,1	3,6	2,9	4,0	4,4	2,0	1,9	
	**Pooled %CV**	**4,2**	**4,6**	**2,6**	**4,2**	**2,4**	**2,3**	**3,3**	**5,3**	**4,7**	**5,2**	**3,4**	**3,3**	**2,9**	**3,7**

Inter-assay variability

	Plates	IL-17F	GM-CSF	IFNγ	IL-10	CCL20/MIP3α	IL-13	IL-17A	IL-22	IL-9	IL-2	IL-5	IL-27	TNFα	
		Obs Conc	Obs Conc	Obs Conc	Obs Conc	Obs Conc	Obs Conc	Obs Conc	Obs Conc	Obs Conc	Obs Conc	Obs Conc	Obs Conc	Obs Conc	
	1	1584,0	3973,9	640,9	71,0	346,4	457,2	732,2	2392,0	512,2	802,2	371,6	3539,9	132,3	
	2	1466,5	3390,0	749,9	71,9	325,5	417,3	683,2	1993,9	454,0	750,2	329,8	3469,4	125,4	
	3	1372,5	3608,7	756,4	68,5	332,3	455,3	707,8	2451,8	486,6	732,5	393,6	3305,0	125,0	
	4	1493,4	4070,2	677,0	75,6	373,8	499,6	743,7	2436,3	518,8	806,3	372,5	4406,3	140,9	
	5	1531,4	3986,9	714,3	66,4	337,1	448,6	728,4	2380,1	451,9	809,4	356,1	4290,8	126,7	
	6	1479,4	3826,9	731,7	70,2	328,6	475,3	741,9	2232,9	487,0	748,4	364,5	3740,9	135,7	
**QC1**	7	1502,0	3654,1	607,1	68,8	338,7	471,7	723,8	2398,8	510,3	761,8	354,0	3799,0	131,7	
	8	1584,9	4169,9	677,1	78,4	348,9	504,2	791,2	2563,2	533,0	826,4	427,9	3749,2	149,2	
	9	1330,5	3180,5	609,8	63,2	319,1	437,0	655,6	2128,3	471,3	721,9	345,3	3530,5	125,3	
	10	1403,1	3599,8	840,4	78,3	327,4	447,3	698,7	2096,7	459,1	749,0	367,9	3079,4	128,6	
	11	1389,3	3705,3	603,6	67,5	328,5	439,5	724,2	2418,1	478,3	735,9	357,4	4223,0	116,7	
	12	1335,1	3442,6	594,1	63,1	325,5	412,6	656,9	2096,3	447,4	694,8	344,4	3555,5	116,2	
	13	1386,4	3679,6	717,2	72,5	393,7	463,5	723,7	2322,1	518,7	761,6	332,2	3371,6	129,1	
	**%CV**	**6,0**	**7,7**	**10,8**	**7,1**	**6,3**	**6,0**	**5,1**	**7,5**	**6,0**	**5,1**	**7,2**	**10,8**	**7,0**	**7,1**
	1	6721,1	15626,4	2917,4	354,1	1781,2	2179,5	3520,0	10074,9	2460,6	3702,5	1626,4	18425,4	675,3	
	2	6809,7	18488,6	3963,5	398,8	1643,1	2193,0	3637,1	11333,0	2625,3	3823,9	1679,7	21145,2	731,0	
	3	6196,5	16254,9	2783,6	304,7	1300,1	1858,9	3220,6	9360,1	2320,6	3427,6	1528,8	16932,2	580,4	
	4	6487,6	17339,5	3050,8	329,0	1464,1	2238,0	3547,2	10087,5	2576,5	3762,9	1734,4	20477,8	703,4	
	5	6207,9	15080,0	3064,6	310,3	1405,2	2081,1	3152,0	10221,0	2040,2	3510,2	1585,1	19352,4	608,9	
	6	5928,7	16335,1	2992,2	321,1	1393,8	2085,5	3230,9	9703,5	2293,2	3475,9	1630,9	18497,1	656,8	
**QC2**	7	6443,6	18850,2	2755,5	311,9	1508,1	2094,5	3528,4	10374,5	2515,6	3494,5	1515,3	15279,3	676,3	
	8	5671,3	15099,6	2737,8	317,0	1441,5	2123,7	3272,6	8749,5	2095,3	3676,1	1710,6	17323,6	631,7	
	9	6230,2	15936,2	2854,2	316,6	1490,8	2149,2	3343,2	9083,0	2463,8	3526,6	1690,1	17722,1	684,9	
	10	6209,9	16675,2	3892,9	388,3	1534,0	2118,3	3648,3	9831,8	2300,2	3682,4	1724,0	16502,8	699,1	
	11	6827,2	16635,8	2831,0	333,8	1429,7	2201,1	3465,5	11154,9	2304,6	3727,4	1774,2	21793,9	683,9	
	12	6191,1	15699,5	2720,9	314,1	1397,0	2030,2	3212,1	9278,7	2144,4	3418,6	1630,6	19127,1	640,0	
	13	6275,2	14275,7	3202,8	326,5	1482,3	2088,6	3273,9	9531,0	2192,5	3500,5	1596,2	17693,0	625,2	
	**%CV**	**5,3**	**8,0**	**13,5**	**9,0**	**8,2**	**4,5**	**5,2**	**7,6**	**7,9**	**3,8**	**4,8**	**10,2**	**6,4**	**7,3**
